# Estimating the national cost burden of in-hospital needlestick injuries among healthcare workers in Japan

**DOI:** 10.1371/journal.pone.0224142

**Published:** 2019-11-07

**Authors:** Hiroyuki Kunishima, Emiko Yoshida, Joe Caputo, Hiroshige Mikamo

**Affiliations:** 1 Department of Infectious Diseases, St. Marianna University, Kanagawa, Japan; 2 Healthcare to All Co. Ltd., Tokyo, Japan; 3 Vista Health Pte. Ltd., Singapore, Singapore; 4 Department of Clinical Infectious Diseases, Aichi Medical University, Aichi, Japan; Tabriz University of Medical Sciences, IR Iran, ISLAMIC REPUBLIC OF IRAN

## Abstract

**Background:**

Needlestick injury (NSI) is one of the most burdensome professional hazards in any medical setting; it can lead to transmission of fatal infectious diseases, such as hepatitis B, hepatitis C and human immunodeficiency virus. In the United States, the annual cost burden was estimated as somewhere between $118 million to $591 million; in the United Kingdom it is approximated to be £500,000 (US$919,117.65) per the National Health Service.

**Method:**

This is the first published paper on the national cost burden of NSIs in Japan. A systematic literature review was conducted to review previous study design in global studies and to extract parameter values from Japanese studies. We conducted abstract searches through PubMed and the Japan Medical Abstracts Society (Ichushi), together with grey literature and snowball searches. A simple economic model was developed to calculate cost burden of NSIs from a societal perspective over a one-year time horizon. We assumed all NSIs are reported and perfect adherence in post NSI management that presented in the labour compensation scheme. Local guidelines were also referenced to extract resource utilization. Lastly, a deterministic sensitivity analysis was conducted and a scenario analysis which considered a payer perspective was also included.

**Result and conclusion:**

The national cost burden of in-hospital NSIs is estimated as ¥33.4 billion (US$302 million) annually, based on an average cost per NSI of ¥63,711 (US$577) and number of NSIs at 525,000/year. 70% of the cost is due to initial laboratory tests, followed by productivity loss, estimated at 20% of the total cost. Cost of contaminated NSIs remains at 5% of the total cost. Change in number of NSIs significantly influences outcomes. Variation in post-exposure management practices suggests a need for NSI specific National guidelines and holistic labour compensation scheme development in Japan.

## Introduction

Needlestick injury (NSI) is one of the most burdensome professional hazards in any medical setting. Infectious disease transmission to healthcare workers, such as hepatitis B (HBV), hepatitis C (HCV) and human immunodeficiency virus (HIV) due to NSI has been reported throughout the world. In Japan 40–50 new HCV cases are reported annually by healthcare workers as the result of injury at work.[1]. Despite an international NSI reporting system developed in 1991 by the University of Virginia, “Exposure Prevention Information Network (EPINet®)”,[2], which is also widely applied in Japan, the reporting rate of in-hospital NSI remains low in Japan, estimated at less than 20.7% [3]. To date, several official reports of the cost burden of NSI have been published by organisations such as the United States (US) General Accounting Office (GAO) (2000) [4] alongside prevention laws, such as the US Needlestick Safety and Prevention Act (2000) and Council Directive 32 (2010) in the European Union (EU), encouraging a NSI free environment. It is based on the consensus that employers must properly consider worker health and safety when designing work processes and by providing suitable equipment, such as safer needle devices, finger shields and sharps bins. In Japan, there are few reports on the burden of NSI and no national regulation exists to tackle NSI. As a result, post exposure management is not standardised–each institution has its own protocol for the management of NSI, as well as its own payment scheme, meaning national level action against NSI has so far not been forthcoming.

Despite the challenge in generalization, our study attempted for the first time in Japan, to estimate the national cost burden of NSIs by using latest available information to select both model structure and parameter values. Guidelines and institutional protocols were collated and carefully assessed by professional medical doctors before being used in the analysis model. We present an overview of the cost burden of NSIs to understand the current situation in Japan, aiming toward a NSI-free work environment for healthcare workers in this country.

## Methods

Our study methodology followed three steps. The first step was a systematic literature review (SLR) of global practices to estimate cost of NSI and to collect parameter values from Japanese studies. In the second step, we developed a cost model to estimate the national cost burden of NSIs in Japan, by reviewing literature retrieved through our search and subsequent discussion with medical authors on the most appropriate structure. Finally, we applied sensitivity analysis to key parameter values, including varying parameters identified and extracted through the SLR between a range of values. The sensitivity analysis also included a scenario analysis where we adopted a payer perspective in addition to the base case societal perspective.

At the initiation of the SLR, a search strategy including a set of search terms and key words were agreed by all authors (S1 Appendix). A PubMed search was conducted in October 2018, to identify all studies on the cost burden of NSIs in English or Japanese with no publishing year restriction. Case studies, discussion papers and editorials were excluded. A search of the Japan Medical Abstracts Society (Ichushi) was also conducted in October 2018 to extract parameter values from studies conducted in Japan, also with no restriction on publication year. Selected articles from both databases were subject to a title and abstract review using a list of inclusion/exclusion criteria (S2 Appendix). The articles, together with additional articles identified through a grey literature search and snowball search, were subject to a full text review and snowball search before the final set of relevant articles was confirmed. Data was extracted from included articles using a data extraction table which was developed and agreed by all authors. Both the review process and data extraction were conducted and cross-checked by two professional researchers.

In developing our model, we also reviewed available NSI management guidelines to identify a common treatment path based on current practice, and also reviewed updated HCV treatment guidelines in order to extract the treatment regimen.

The SLR on parameter values identified a limited number of multi-setting studies, with mostly single setting studies found in the published literature. Almost all studies were reports or summary of reports, so meta-analysis was not considered possible and the SLR therefore is presented as a qualitative synthesis of published data. We applied parameter values from the latest and the most reliable sources to the base case study, while values from other less reliable sources were applied only to sensitivity analysis.

Monetary value are presented in the original currency year with value equivalent to United States Dollar in the same year by applying OECD annual exchange rate [5] in bracket.

## Model structure

**Fig 1:**
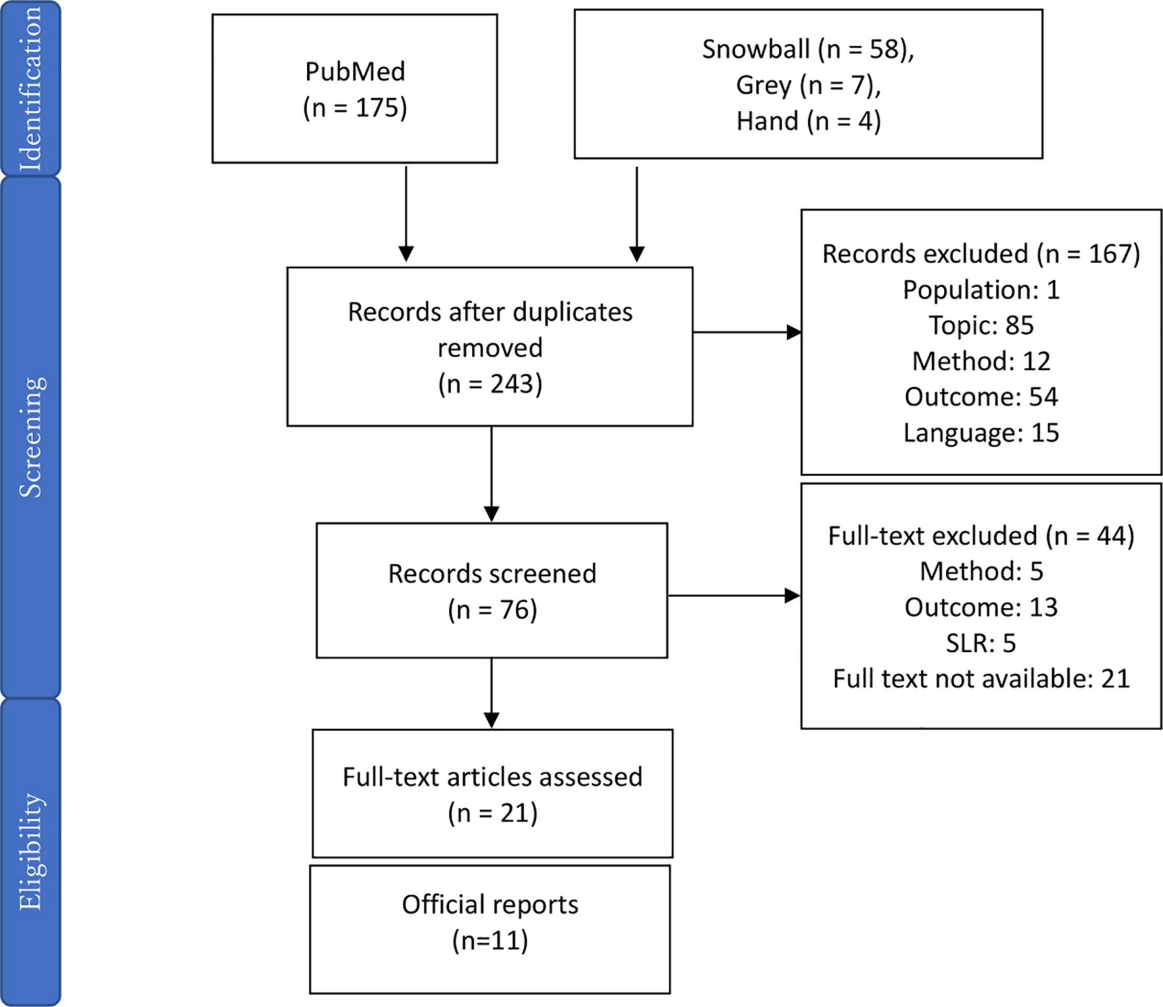
Result of SLR for model design.

The first SLR identified 244 articles, including 175 articles from the PubMed search. 21 articles met our inclusion criteria after title, abstract and full-text review. (Fig 1) Many of these articles were based on analysis from a hospital perspective, applying hospital fees rather than reimbursement or out of pocket fees, as recommended in CDC guideline [6], while six articles presented either a payer or societal perspective, as summarized in Table 1.

**Table 1:** List of articles with payer or societal perspective.

Author	Year	Country	Population	Outcomes	Perspective	Time horizon	NSI	Resource utilization	Infectious disease	Productivity loss	Emotional distress	Other cost included
McCormick et al. [10]	1991	USA	HCW	Burden	Societal*	Not reported	Report (Retrospective)	Compensation and protocol	PEP and lab	Included	Not included*	Compensation
Lee et al. [17]	2005	USA	Acute care nurses	Cost Burden	Societal	1 year	Literature	Survey and guideline	PEP, lab, clinic visit	Included	Included (counselling)	Not known
Glenngard et al. [20]	2009	Sweden	HCW*	Cost Burden	Healthcare	Not reported	Report (Retrospective)	Literature and KOLs	PEP, lab, clinic visit	Not included	Included (counselling)	Not known
Jason [[23]	2013	USA	Community HCW	Cost Burden	Payer*	Not reported	Clinical database	Claim database	PEP, lab, clinic visit	Not included	Not included	Tetanus toxoid
Oh et al. [24]	2013	South Korea	HCW	Burden	Societal*	Not reported	Prospectively corrected	Prospectively corrected	PEP, lab, clinic visit	Included	Not included*	Surgery, HAV, venereal disease
De Jager et al. [27]	2018	South Africa	HCW	Cost-Effectiveness	Payer	45 years	Literature	Guidelines	PEP and lab	Included	Not included*	Not known

HAV: Hepatitis A virus; HCW: healthcare worker; PEP: post exposure prophylaxis; NSI: needlestick injury

*not explicitly stated–based on our best guess

Centers for Disease Control and Prevention (CDC) provides a standard model of calculating the cost of NSI [6] from a hospital perspective that includes work time for laboratory technologist and consultant, rather than what payers in healthcare or the social system pay. National Health Service (NHS) Scotland lists in its definition of burden of NSI [7] laboratory testing, post exposure prophylaxis, treatment of blood borne viral infection, productivity loss, counselling injured workers and legal consequences. Despite the recommendations, the identified articles (Table 1) show a variation in analysis structure in terms of perspective, time horizon and data source. None of the studies describe in detail the structure of their economic model except De Jager (2018) [8], who developed a Markov simulation model with one-year cycle and seven health states over 45 years. All studies take the number of NSIs from spontaneous reporting systems, either via retrospective review of reports, prospective collection of data from reports, or a summary of reports found in literature. They tend to ignore unreported cases, which could be as many as half of the injuries in the US [9] and 74% in the UK [10]. Treatment adherence was assumed to be 100% and resource utilization estimates were often taken from protocols and guidelines, while some extracted real-world resource utilization estimates from reports. As a result of these structural variations, the outcome widely varies from US$60/NSI to US$1,687/NSI.

In Japan, NSI management fees are payable by the labour compensation scheme, however not all hospitals follow this rule. A study by Arise et al in 2013 reported only 62.3% of hospitals paid medical costs using labour compensation scheme to all post NSI medical services, based on a survey of 159 medical settings in one prefecture in Japan [11]. In our study we adopted a societal perspective, applying 100% of labour compensation scheme coverage for medical expenses, and also included productivity loss. Additional analysis adopting a payer perspective was conducted in a sensitivity analysis. Time horizon was set to one year, since almost all NSI management guidelines recommend six months to one year follow up to monitor infectious transmission status (Table 2), and standard treatment of acquired HCV lasts for twelve weeks (Table 3).

**Table 2:** Guidelines for NSI management.

Publisher	Year	Source test conducted	Follow up period				
			No infection or immune	Unknown	HBV	HCV	HIV
Drs’ association [12]	2007	Yes; If not known	1,3,6 mth	1,3,6 mth	1,3,6 mth	1,3,6 mth	1,3,6,12 mth
Labour compensation [13]	2010	Yes; If not one year	3 mth	3 mth	2w, 1,2,3,6 mth	1,2,3,6 mth	1,2,3,6 mth
Drs’ research (MHLW) [14]	2013	Yes	Not reported	1,3,6,12 mth	1,3,6,12 mth	1,3,6,12 mth	6,12w, 6 mth
Specialist Drs’ Association [15]	2015	Yes; if not known	Not reported	Not reported	1,2,3,4,5,6,12 mth	Every 2-4w up to 6mth	Not reported
Aki Hospital [16]	2016	Yes; if not in one year	3 mth	1,3,6 mth	1,3,6 mth	1,3,6 mth	6w, 3,6 mth (if with HCV, 12mth)
Saitama Gov.[17]	2017	Yes	Not reported	Not reported	Not reported	1,2 w	6,12 w, 6 mth
Hokkaido Uni Hospital [18]	2018	Yes	No need	1,3,6 mth	1,3,6 mth	1,3,6 mth	1,3,6 mth (if with HCV, 12mth)
Tsukuba Uni [19]	2018	Yes	Not reported	Not reported	1,6 mth	3,6 mth	Not reported
Research centre [20]	2018	Not reported	Not reported	Not reported	Not reported	Not reported	6,12 w, 6 mth (if with HCV, 12mth)
Kagawa Uni Hospital [21]	Not reported	Yes; If not in 3 mth	Not reported	1,2,6 mth	2,6 mth	1,6 mth	1,3,6,12 mth
AIDs research centre [22]	Not reported	Not reported	Not reported	Not reported	Not reported	Not reported	6,12 w, 6 mth (if with HCV, 12mth)
Nagoya Uni Hospital [23]	Not reported	Not reported	Not reported	1,3,6,12 mth	1,3,6,12 mth	1,3,6,12 mth	6w, 3,6,12 mth
Kagoshima Uni Hospital [24]	Not reported	Yes	3,6 mth	1,3,6,12 mth	1,3,6,12 mth	1,3,6,12 mth	1,3,6,12 mth
Maizuru Med Centre [25]	Not reported	Yes; If not in 6 mth	1,3,6 mth	1,3,6 mth	1,3,6,8 mth	1,3,6,8 mth	1,3,6,8 mth
Professional Association [26]	Not reported	Yes	Not reported	Not reported	(up to 6 mth)	(up to 6 to 12 mth)	4-6w, 3mth, 6-12mth

Mth: Months, w: week

**Table 3:** HCV treatment regimen, guidelines for HCV treatment, 2018 [27].

Geno type	Drug	Tablet	Price/tab	Dose	# visit*
1	SOF/LDV	ledipasvir 90mg /sofosbuvir 400mg	¥54,685.9 (US$495.24)	X1/day x12weeks	6
1	EBR	elbasvir 50mg	¥25,982.5 (US$235.30)	X1/day x12weeks	6
	GZR	grazoprevir 100mg	¥9,281.9 (US$84.06)	X1/day x12weeks	
1	GLE/PIB	glecaprevir 100mg /pibrentasvir 400mg	¥24,180.2 (US$218.98)	X3/day x8weeks	4
1	BCV/DCV/ASV	ombitasvir 12.5mg /paritaprevir 75mg /ritonavir 50mg	¥22,201.1 (US$201.06)	X2/day x12weeks	12*
2	SOF	sofosbuvir 400mg	¥42,238.0 (US$382.51)	X1/day x12weeks	6
	RBV	ribavirin tablet200mg (generic)	¥345.6 (US$3.13)	X3/day x12weeks	
2	GLE/PIB	glecaprevir 100mg /pibrentasvir 400mg 100/400mg	¥24,180.2 (US$218.98)	X3/day x8weeks	4
2	SOF/LDV	ledipasvir 90mg /sofosbuvir 400mg 90/400mg	¥54,685.9 (US$495.24)	X1/day x12weeks	6

*Number of visits was hypothesised by medical doctors as being once every two weeks with a set of laboratory tests in every visit, except ombitasvir 12.5mg /paritaprevir 75mg /ritonavir 50mg which requires weekly monitoring (S2 Appendix: Inclusion-Exclusion criteria).

A shorter time horizon reduces uncertainty around lifetime costs, e.g. lifetime treatment of HBV and HCV, which were also not included in the previous studies.

## Parameter values

### Needlestick injuries and reporting

**Fig 2:**
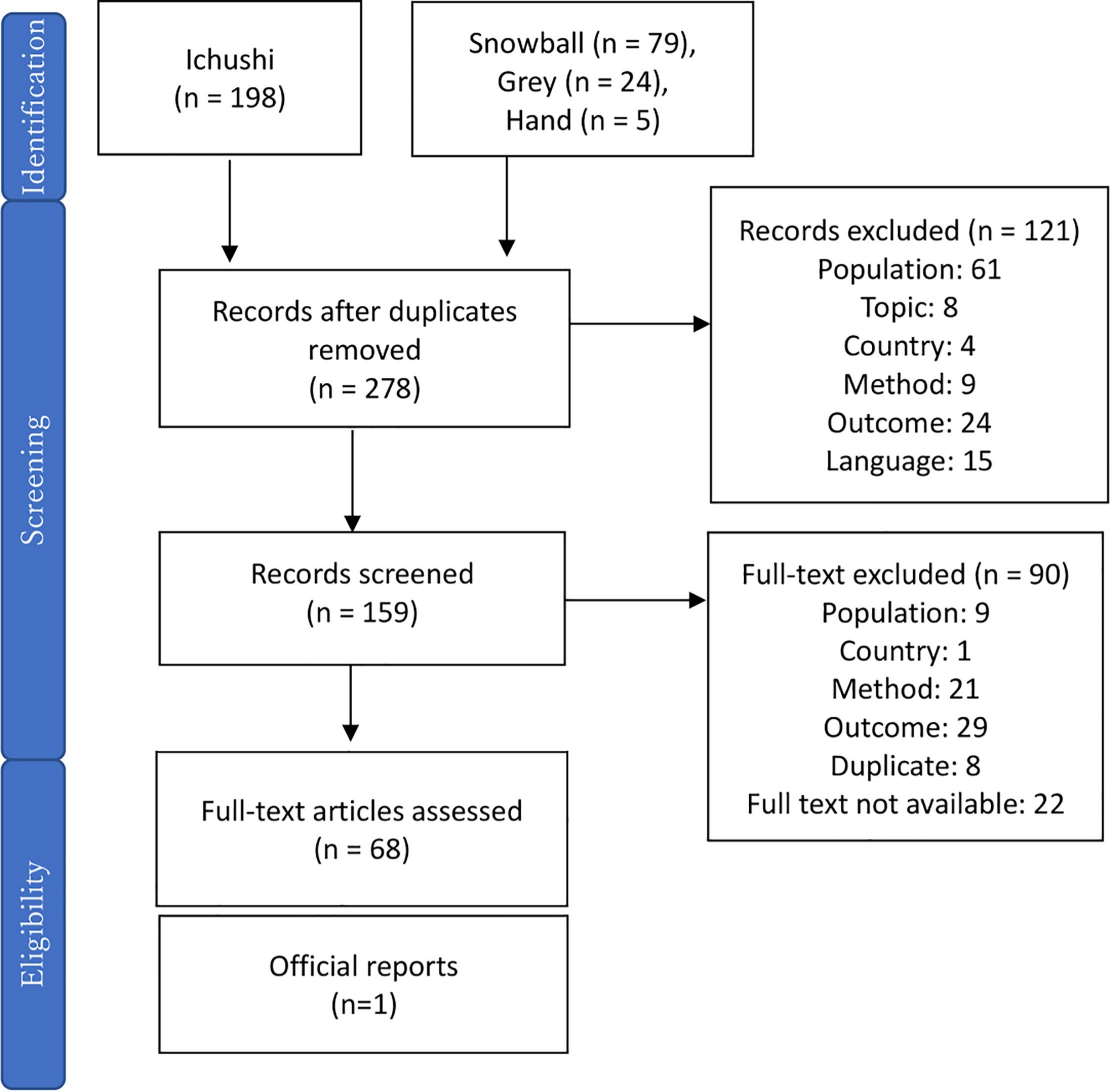
Result of SLR for parameter value.

Parameter values, including number of NSIs, resource utilization and other costs were the subject of a second SLR. 304 articles were identified, including 198 articles from the Ichushi search. 68 articles met our inclusion criteria after title, abstract and full-text review. (Fig 2) Only one publication, Kimura 2003, estimated the national number of NSIs in Japan. Six studies reported the rate of NSI in a multi-centre study (Table 4), and five studies reported the NSI reporting rate in a multi-centre study (Table 5).

**Table 4:** Rate of needlestick injuries in multi-centre studies.

Author	Year	Setting	Population	Method	% NSI
Kimura et al [28]	1997	155 training hospitals	HCW	Postal survey to hospitals with EPINet	4.00 NSIs/100beds
Kidouchi et al [29]	2000	198~225 AIDS hospitals	HCW	Postal survey to hospitals with EPINet	4.00 NSIs/100beds (3.99, 4.25, 4.29 NSIs/100beds over three years)
Kimura [30]	2003	921 AIDS hospital years	HCW*	EPINet report	4.2 NSIs/100beds. Estimated as 30–40 NSIs/100beds with reporting rate (Hypothesis) at 10–15%
Kidouchi et al [31]	2004	101 AIDS hospitals	HCW	Postal survey to hospitals with EPINet	5.3 NSIs/100beds
Maeda et al [32]	2010	All medical settings in Kumamoto city (response rate at 40.3%)	HCW	Survey	4.9 NSIs/100beds
Yoshikawa et al [33]	2013	110 AIDS hospitals	HCW*	EPINet report	6.2 (95CI: 5.7–6.7) NSIs/100beds (4.8 in <400beds hospitals, 6.7 in 400-799beds hospitals, 7.6 in 800 or more beds hospitals)

NSI: needlestick injury

*not explicitly stated–based on our best guess

**Table 5:** Needlestick injuries reporting rate in multi-centre studies.

Author	Year	Setting	Population	Method	% reporting
Kanda et al [34]	1998	8 hospitals in a region	HCW	Question sheet	34.7%
Kidouchi et al [29]	2000	198~225 training hospitals	HCW	Hypothesis; all HCV is reported and HCV prevalence in patient is 7–10%	12~22%
Kidouchi et al [35]	2003	921 AIDS hospital years	HCW*	Hypothesis; all HCV is reported and HCV prevalence in patient is 7–10%	17%
Kou et al [36]	2005	35~37 hospitals that introduced EPINet	HCW	Hypothesis; all HCV is reported. HCV prevalence in patient is not provided	9~16% in medical doctors, 10~32% in nurses
Hiramitsu and Yoshikawa [3]	2017	22 hospitals	HCW	Proportion of HCV positives in total blood tests was 2.2%	No more than 20.7%

HCW: health care workers

*not explicitly stated–based on our best guess

Number of NSIs reported from a single setting varied not only in terms of the number itself, but also in how they were reported–the most commonly used outcomes were number of NSIs per bed or per nurse; 9–23.5 NSIs/100,000nurses, 12–36 NSIs/100beds. Kimura (2003) estimated the number of NSIs in Japan as 525,000 [30], which was used for our base case analysis; calculated number of NSIs estimated from the rate of NSI (Table 4) and the reporting rate (Table 5) were used for the deterministic sensitivity analysis.

Under reporting is widely recognised as a key challenge in NSI studies. There are two approaches used to estimate reporting rate. The simplest way is to ask healthcare professionals about their experience in NSI, and whether and how often NSI is reported. Estimated reporting rates in studies using this simple technique ranged from 11.9% to 90% [37] [38] [34] [39] [40] [41] [42] [43] [31] [44] [45] [46] [47] [48]. Other studies adopted the ratio of HCV prevalence in source patient vs. prevalence in in-hospital patients and estimated the reporting rate by hypothesizing all HCV contaminated NSIs are reported. HCV prevalence among patients were estimated as either 7% or 10% (no source provided), and reporting rates ranged from 7% to 40.7% [37] [49] [50] [29] [35] [36] [51] [52] [3]. Kidouchi (1998) compared estimated reporting rates using the two methods, resulting in a 7% rate using the HCV prevalence method vs 12% using simple surveys in 1992, and 10% vs 39% respectively in 1995 [37]. Reporting rates from single setting studies were excluded from our analysis.

### Prevalence of HBV and HCV

Prevalence of HBV and HCV are difficult to estimate because not all hepatitis carriers present with symptoms and may therefore not be recorded. Tanaka in 2004 [53], 2011 [54] and 2018 [55] referenced the prevalence of hepatitis among first time blood donors as a representative sample of Japanese general population and reported the latest estimates in numbers of HBV and HCV in Japan in 2011. We calculated prevalence of hepatitis using 2011 Japanese population data published by Japan’s official statistics bureau as the denominator [56]. HBV prevalence in the general population in 2011 was estimated at 0.88–1.0% (median 0.942%). HCV prevalence in the general population in 2011 was estimated at 0.78–1.25% (median 1.013%). HCV prevalence in hospital patients was thought to be higher than that of the general population. Kidouchi (2000) [29] and Kidouchi (2003) [35] estimated reporting rates by using HCV prevalence among patients at 7 to 10% using unknown sources. Four studies surveyed and reported HCV prevalence among patients in their settings (Table 6).

**Table 6:** HCV prevalence among patients.

Author	Year	Setting	Year of survey	% HCV+ among patients
Kidouchi et al [57]	1997	Five hospitals in a region	1992 financial year and 1995 financial year	5% and 5.3%
Kidouchi et al [37]	1998	A city hospital	1992–1994, 1995 and 1996	5%, 8%, 7%
Yukawa [51]	2005	A city hospital	2000	6%
Suewaka [58]	2007	A university hospital	2005	9.86%

In our study, we applied the HBV prevalence from the general population and a HCV prevalence of 5% for the base case analysis, varying the figure between 0.78% and 9.86% in the sensitivity analysis.

Prevalence of the HBV antibody in those injured is another important parameter which determines amount of post exposure prophylaxis required. Although vaccine administration is widely recognized as effective against HBV, not everyone obtains immune status and the vaccine is not mandatory for HCW in Japan. Table 7 summarizes the reported prevalence of HBV antibody among healthcare workers.

**Table 7:** Prevalence of HBV antibody among HCW.

Author	Year	Setting	Population	% HBV antibody positive
Kidouchi et al [35]	2003	921 AIDS hospital year	HCW*	56%
Yamazaki et al [59]	2005	A community hospital	HCW	56%
Hatanaka et al [60]	2006	A hospital	HCW	Approximately 40%
Nagao et al [61]	2007	A university hospital	HCW	53.3%
Sumimoto et al [62]	2009	A hospital	HCW	50% in all HCWs and 51.6% of injured
Oishi et al [63]	2011	A hospital	HCW	66%
Otsu et al [64]	2013	A city hospital	HCW*	Increased from 73.0% (2009 financial year) to 88.4% (2011 financial year) (all employee received vaccine in 2009)

HCW: health care worker

*not explicitly stated–based on our best guess

Among the five articles, only one study, Kidouchi (2003) [35], was a multi-centre study, therefore the point estimate from this study is used for our base case analysis. The remaining studies were single setting studies and their estimates were applied to our deterministic sensitivity analysis.

### Resource utilization

There were only a limited number of reports regarding resource utilization (Table 8).

**Table 8:** Resource utilization.

Author	Year	Reference	Initial laboratory test	Number of follow ups
Takahashi et al [65]	1999	Hospital protocol	Both source patient and injured are included	5: at 1, 2, 3, 6, 12 months
Kidouchi et al [35]	2003	Not reported	HIV-ab 23%, HCV-ab 85%, HBs-ag 67%	Not reported
Suewaka [58]	2007	Question sheet	Not reported	Average follow up days are 3.65 (in 2005/6) and 3.75 (in 2005/6). Adherence of follow up at 21.7% (in 2005/6) and 18.8% (2004/5)
Horikawa et al [46]	2007	Question sheet	Not reported	3; at 1, 3, 6 months. Survey showed 36% with no follow up, 27.8% with once, 19.5% with twice and 4% complete 6 months follow ups.
Sumimoto et al [62]	2009	Not reported	Not reported	3; at 1, 3, 6 months.
Arise et al [11]	2013	Question sheet	[Injured] HBs-ag 93.7%, HBs-ab 86.8%, HCV-ab 90.6%, HIV-ab 30.8%. Previous test result 13.2%, test at injury 64.8%, both 10.7% [source] HBs-ag/HCV-ab 94.3%, HIV-ab 37.1%. Previous test result 50.9%, test at injury 42.8%	Not reported

HCW: health care worker, HBs-ab: Hepatitis B surface antibody; HIV-ab: HIV antibody, HBs-ag: Hepatitis B surface antigen, HCV-ab: Hepatitis C virus antibody

Resource utilization usually includes, but is not limited to, laboratory tests, clinic visits, post exposure prophylaxis (PEP) and treatment for infectious disease. Studies extracted resource utilization from hospital protocols, surveys or both, with a high degree of variation especially in laboratory tests and number of follow ups. Protocol adherence is not 100%, with Kidouchi (2003) reporting a maximum of 85% of source patients undergoing laboratory tests [35]. Arise (2013) reported laboratory tests conducted at time of injury in only 4% of source patients and 75% of injured [11].

We referred to hospital protocols and professional guidelines for post-NSI management in the absence of data on current usual practice (Table 2). We also found that resource utilization varied widely according to setting and professional organization. Some hospital and clinical guidelines suggested there is no follow up for infection-free NSI, while others suggest once- or twice- monthly laboratory check-ups over 6 months for contaminated NSI [15] [18] [21] [23]. Both estimates were considered to be extreme, and so we adopted one follow up for infection-free NSI and three for contaminated NSI in the base case analysis, and 1–3 times and 2–6 times respectively in sensitivity analysis.

Recommendations for laboratory testing at time of injury also differ between protocols. Whilst all protocols recommend checking the infectious status of both source patients and injured HCWs, some accept blood test results conducted within 3 months [21], 6 months [25] or even one year [13] [16] prior to injury. It was not clear if the initial laboratory tests could be reimbursed via the labour compensation scheme, and as a result, costs of initial laboratory tests are assumed to be covered by hospitals or individuals. In our analysis, we assume initial laboratory tests are conducted for both source patients and injured HCWs and covered 100% by the labour compensation, as this makes most sense from a medical practice perspective.

Finally, a productivity loss estimate of four hours was applied at each follow up, based on best available information. Average annual salary for Japanese population published by Ministry of Health, Labour and Welfare was ¥4,320,000 [66] (US$39,122 in 2018 value). By applying number of annual holidays and weekends for government officers as 123 days and daily working hours at eight hours, an average hourly productivity is estimated as ¥2,231 (US$20.2 in 2018 value).

Table 9 shows the final set of parameter values; a detailed calculation of cost of post exposure medical fees is presented in S3 Appendix.

**Table 9:** Parameter base case values and values for the sensitivity analysis.

Parameter	Value	Reference	Values for sensitivity analysis	Reference
Number of NSIs	525,000	Kimura, 2003 [30]	NSI/100beds: 4.0–6.2	Table 4
			Reporting rate: 12%-34.7%	Table 5
			Number of beds in Japan: 1,653,544	MHLW (2018) [67]
HBV prevalence	30%	JRGOICP [68]	Not applied	Not applicable
HCV prevalence	0.8%	JRGOICP [68]	Not applied	Not applicable
HIV prevalence	0.1%	JRGOICP [68]	Not applied	Not applicable
HB-ab in HCW	56%	Kidouchi et al (2003) [35], Yamazaki et al (2005) [59]	40%, 50%, 53.3%, 66%, 73%	Table 7
HCV transmission	1.8%	JRGOICP [68]	Not applied	Not applicable
Test at injury	100% both source patients and injured	Guidelines and protocols (Table 2)	Not applied	Not applicable
HBV prophylaxis	HBIG (iv) and vaccine (x3)	Labour compensation scheme [69]	Not applied	Not applicable
HIV prophylaxis	Truvada® and Isentress®	Labour compensation scheme [70]	Not applied	Not applicable
Number of follow ups	1 for no-infection. 3 for infection	Guidelines and protocols (Table 2)	1 to 3 for no infection. 2 to 6 for infection	Guidelines and protocols (Table 2)
HCV treatment	(see Table 3)	HCV treatment guideline [27]	Not applied	Not applicable
Productivity loss	4 hours	assuming half a day off at each follow up visit	Not applied	Not applicable
	¥2,231 (US$20.20)/hr	average salary of ¥4,320,000 [56] (US$39,122)/(365–123 (bank holidays)) /8 hours (working hours /day)		

HCW: health care worker, NSI: needlestick injury, MHLW: Ministry of Health, Labour and Welfare

## Results

**Fig 3:**
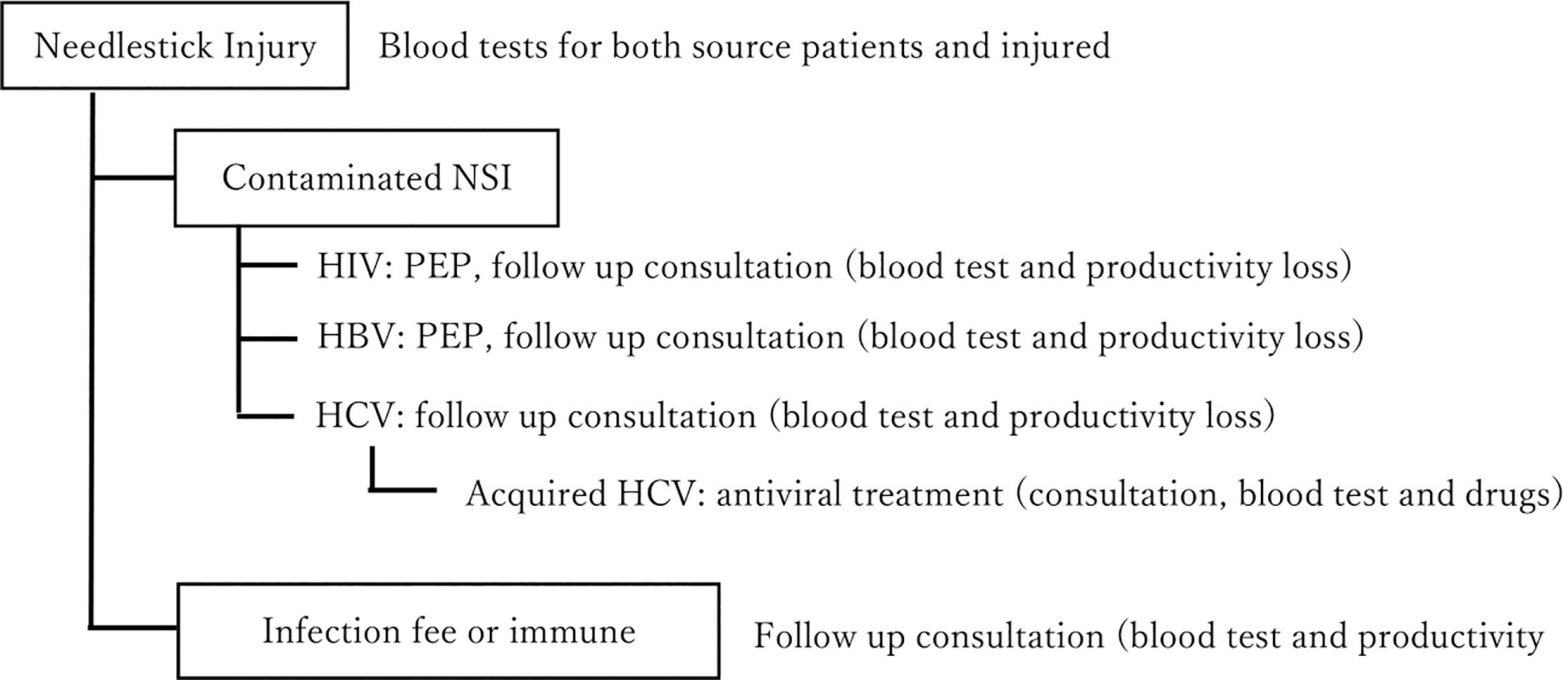
Model structure.

Out of 525,000 needlestick cases, 497,854 cases (94.83%) were estimated to have no risk of HBV, HCV or HIV infection, based on needles not being contaminated with infectious blood and/or injured HCWs being immune. Despite a high HBV antibody prevalence among healthcare workers (56%), 2,130 cases (0.41%) face a risk of HBV infection. There is no approved vaccine or post exposure prophylaxis against HCV, and so a potential 24,489 (4.66%) healthcare workers face a risk of HCV infection, with an estimated 449 HCWs (0.09%) potentially acquiring HCV. HIV contaminated NSIs are estimated at 79 (0.01%) (Table 10). Fig 3 shows our model in which each status associated with unique set of costs.

**Table 10:** Cost of needlestick injury by infectious status.

Case	# cases	% cases	Cost/NSI	Cost breakdown*	Total cost	% of total cost
No infection or immune	497,854	94.83%	¥57,736 (US$522.86)	#1+#2+#5+#8	¥28,743,927,771. (US$260,307,433.88)	85.94%
HBV+	2,130	0.41%	¥147,271 (US$1,333.70)	#1+#2+#3+#6+#8	¥313,639,108. (US$2,840,342.21)	0.94%
HCV+ not acquired HCV	24,489	4.66%	¥103,367 (US$936.10)	#1+#2+#6+#8	¥2,531,312,260 (US$22,923,777.29)	7.57%
HCV+ acquired HCV	449	0.09%	¥4,089,623 (US$37,035.97)	#1+#2+#4+#6+#7+#8	¥1,835,729,403 (US$16,624,520.28)	5.49%
HIV+	179	0.01%	¥302,953 (US$2,743.57)	#1+#2+#4+#6+#8	¥23,783,167 (US$215,382.37)	0.07%

*Each item number is found in the Table 11

Cost per NSI with no infection contamination or immune case is estimated at ¥57,736 (US$522.86). The cost per NSI increased to ¥147,271 (US$1,333.70) with HBV contaminated NSI, ¥103,367 (US$936.10) with HCV and ¥4,089,623 (US$37,035.97) for acquired HCV case, and ¥302,953 (US$2,743.57) with HIV contaminated NSI. Total cost of in-hospital NSIs in Japan is calculated as ¥33,448,391,709 (US$302,911,456.03) at an average cost per NSI of ¥63,711 (US$576.97).

Table 11 shows a breakdown of costs for each post exposure management.

**Table 11:** Cost of each items for post exposure management.

#	Items	Resource utilization	Cost/case	# cases	Total cost	% of total cost
1	Test at injury (source)	x1	¥17,460 (US$158.12)	525,000	¥9,166,500,000 (US$83,012,597)	27.40%
2	Test at injury (injured)	x1	¥17,460 (US$158.12)	525,000	¥9,166,500,000 (US$83,012,597)	27.40%
3	HBV prophylaxis (HBIG + vaccine)	1000iu & x3	¥43,904 (US$397.60)	2,130	¥93,501,263 (US$846,755)	0.28%
4	HIV prophylaxis	Table	¥199,586 (US$1,807.47)	79	¥15,668,413 (US$141,894)	0.05%
5	Follow ups (no infection)	x1	¥13,890 (US$125.79)	497,851	¥6,915,196,509 (US$62,624,603)	20.67%
6	Follow ups (with infection)	x3	¥41,670 (377.37)	27,149	¥1,131,160,473 (US$10,243,885)	3.38%
7	HCV treatment	Table	¥3,986,256 (US$36,099.87)	449	¥1,789,330,604 (US$16,204,329)	5.35%
8	Productivity loss (every follow up)	4 hrs	(¥2,231 (US$20.20)/hr)	579,291	¥5,170,584,447 (US$46,825,249)	15.46%

HBIG: hepatitis B immune globulin, iu: international unit, hr: hour

## Sensitivity analysis

Deterministic sensitivity analysis was conducted to see how sensitive the outcome was to parameters from less reliable sources for 1) number of NSIs, 2) HBV and HCV prevalence, and 3) number of follow ups. We used parameter values from multi-center survey (Table 4 and Table 5). The number of NSIs were varied from 4.0 /100beds [28] [29] to 5.3 /100beds [31] and the reporting rate from 12% [29] to 34.7% [34], resulting in the annual number of in-hospital NSIs in Japan varying widely between 190,610 and 854,331, together with cost of total NSIs between ¥12,577,211,505 (US$113,900,288.03) and ¥56,372,110,068 (US$510,510,582.65), respectively (Table 12).

**Table 12:** Cost of NSIs in Japan with reported number of NSIs per 100 beds and rate of reporting rate.

Rate of reporting rate	Reported number of NSIs per 100 beds				
	4.00 [Kimura, 1997 and Kidouchi, 2000]	4.20 [Kimura, 2003]	4.90 [Maeda, 2010]	5.30 [Kidouchi, 2004]	6.20 [Yoshikawa, 2013]
12.0% [Kidouchi, 2000]	¥36,369,103,270 (US$329,361,666)	¥38,187,558,433 (US$345,829,750)	¥44,552,151,505 (US$403,468,041)	¥48,189,061,832 (US$436,404,208)	¥56,372,110,068 (US$510,510,583)
17.0% [Kidouchi, 2003]	¥25,672,308,190 (US$232,490,588)	¥26,955,923,600 (US$244,115,117)	¥31,448,577,533 (US$284,800,970)	¥34,015,808,352 (US$308,050,029)	¥39,792,077,695 (US$360,360,411)
20.7% [Hiramatsu, 2017]	¥21,083,538,127 (US$190,934,299)	¥22,137,715,034 (US$200,481,014)	¥25,827,334,206 (US$233,894,517)	¥27,935,688,019 (US$252,987,947)	¥32,679,484,097 (US$295,948,164)
22.0% [Kidouchi, 2000]	¥19,837,692,692 (US$179,651,818)	¥20,829,577,327 (US$188,634,409)	¥24,301,173,548 (US$220,073,477)	¥26,284,942,818 (US$238,038,659)	¥30,748,423,673 (US$278,460,318)
34.7% [Kanda, 1998]	¥12,577,211,505 (US$113,900,288)	¥13,206,072,081 (US$119,595,302)	¥15,407,084,094 (US$139,527,853)	¥16,664,805,245 (US$150,917,882)	¥19,494,677,833 (US$176,545,446)

US$ in 2018 value

We could not find generalizable estimates for the prevalence of HBV and HCV among in-hospital patients. Sensitivity analysis was therefore conducted by varying those parameter values using latest Japanese population estimates calculated from first time blood donations (0.88% to 1.00% in HBV and 0.78% to 1.25% in HCV), and reported HCV prevalence among in-hospital patients (5.00% [57] [37] to 9.86% [58]) taken from Japanese single setting surveys. An increase in prevalence of HCV led to an increase in number of post exposure HCV treatments–the most expensive post exposure management cost at ¥3,986,256 (US$36,099.87) /case (Table 13).

**Table 13:** Cost of NSIs in Japan with prevalence of HBV and HCV among in-hospital patients.

HBV prevalence	HCV prevalence				
	0.78%	1.01%	1.25%	5.00% [Kidouchi, 1997 &1998]	9.86% [Suewaka, 2007]
0.88%	¥31,180,112,373 (US$282,369,727)	¥31,376,043,131 (US$284,144,093)	¥31,579,514,191 (US$285,986,744)	¥34,628,964,882 (US$313,602,826)	¥38,315,411,940 (US$346,987,602)
0.94%	¥31,192,008,651 (US$282,477,461)	¥31,387,939,409 (US$284,251,826)	¥31,591,410,469 (US$286,094,477)	¥34,640,861,161 (US$313,710,560)	¥38,327,308,219 (US$347,095,335)
1.00%	¥31,203,871,086 (US$282,584,888)	¥31,399,801,844 (US$284,359,253)	¥31,603,272,904 (US$286,210,905)	¥34,652,723,595 (US$313,817,987)	¥38,339,170,653 (US$347,202,763)

US$ in 2018 value

Varying the number of follow ups where there is no infectious contamination from one to three changes the outcome significantly, since this applies to 95% of NSIs, while NSIs with related infection remains at 5%. Varying the number of follow ups for contaminated NSI from two to six has limited impact on outcomes (Table 14).

**Table 14:** Cost of NSIs in Japan with number of follow ups after NSI with risk of infection and without risk of infection.

Number of follow ups without risk of infection	Number of follow ups with risk of infection				
	2	3	4	5	6
1	¥34,399,236,660 (US$311,522,388)	¥34,641,556,348 (US$313,716,856)	¥34,883,876,035 (US$315,911,323)	¥35,126,195,723 (US$318,105,791)	¥35,368,515,410 (US$320,300,258)
2	¥38,842,867,386 (US$351,764,283)	¥39,085,187,073 (US$353,958,750)	¥39,327,506,761 (US$356,153,218)	¥39,569,826,449 (US$358,347,685)	¥39,812,146,136 (US$360,542,153)
3	¥43,286,498,112 (US$382,950,093)	¥43,528,817,799 (US$394,200,645)	¥43,771,137,487 (US$396,395,112)	¥44,013,457,174 (US$398,589,580)	¥44,255,776,862 (US$400,784,047)

US$ in 2018 value

Finally, we re-calculated costs based on a scenario analysis where we adopted a payer perspective. The national cost of in-hospital NSIs was estimated to be ¥21,097,052,361 (US$191,056,685.30) with cost per NSI reduced to ¥40,185 (US$363.92) (Table 15).

**Table 15:** Cost of needlestick injuries from the societal and a payer perspective.

Status of infection	Number of cases	Cost/case–societal perspective	Cost/case–payer perspective	Total cost–payer perspective
No infection or immune	497,854	¥57,736 (US$522.86)	¥35,510 (US$321.58)	¥17,678,806,914 (US$160,100,766)
HBV+	2,130	¥147,271 (US$1,333.70)	¥99,936 (US$905.03)	¥212,831,228 (US$1,927,418)
HCV+ not acquired HCV	24,489	¥103,367 (US$936.10)	¥56,250 (US$509.41)	¥1,377,485,156 (US$12,474,622)
HCV+ acquired HCV	449	¥4,089,623 (US$37,035.97)	¥4,027,777 (US$36,475.89)	¥1,807,968,343 (US$16,373,114)
HIV+	179	¥302,953 (US$2,743.57)	¥254,262 (US$2,302.62)	¥19,960,720 (US$180,766)

HBIG: hepatitis B immune globulin

US$ in 2018 value

## Discussion

### Comparison to the previous studies

Our study uses Kimura’s estimate of the total number of NSIs of 525,000 (450,000 to 600,000 [30]), which is calculated by Kimura using a rate of NSI of 4.0 per 100 beds and reporting rate of 10% to 15%. This is slightly higher than the US GAO estimate; the annual total number of in-hospital NSIs in the US is estimated as 236,000 with an estimated under reporting rate of 50% [4] [6]. We hypothesise that differences in total number of NSIs are attributable mainly to differences in number of beds per capita (1,664,456 [2016] vs. 897,961 [2016] [71]) and in length of stay (16.5 [2015] vs. 6.1 [2015]) in Japan and US respectively [72]. It can also be explained by estimated NSI reporting rates at 50% and 15% respectively. The national cost of NSIs in Japan was estimated as ¥33,448,391,709 with an average cost per NSI of ¥63,711, which is equivalent to US$303 million and US$577 respectively using an exchange rate of ¥110.42/US$ (2018 OECD exchange rate). Both cost per NSI and the total National cost of NSIs are lower in Japan compared to that presented in GAO’s report; $118 million, $354 million, or $591 million using a cost of NSI at $500, $1,500, or $2,500 /NSI.

The UK Health and Safety Executive (HSE) reported the number of estimated NSIs at 85,000, based on 40,000 reported NSIs a year (adjusted by non-reported rate of approximately 50%) from NHS Employers estimates (2005) [73]. HSE’s figure is very small compared to our estimate in Japan. However, the figure presented by HSE could be 400,000, similar to that of Kimura’s study, were a non-reported rate of 10% provided by Elder and Paterson (2006) to be used instead. HSE assumed the majority of NSIs lead to very minor injuries and used £350 (US$636.36 in 2005 value) as the cost of typical NSI based on the 2005 (Q3) HSE Economic Analysis Unit assumption. This cost includes human costs (pain and grief), resource costs and costs of lost output [74]. NHS Employers also shows a similar figure; “an injury involving a known patient that posed a low risk of cross-infection cost the organisation between £330 and £404” [10] (US$503.82 and US$616.79 in 2015 value, respectively). In our analysis estimated cost per NSI was ¥63,711 (US$576.97 in 2018 value). Considering the UK as one of the more successful healthcare cost management countries, our estimate would be recognised as reasonable. It is worth noting that in addition to medical costs and productivity loss, UNISON, the UK’s largest union, had successfully negotiated a deal with NHS for an immediate claim for needlestick injuries at £2,000 [75] (US$3,267.97 in 2003 value).

We have also compared our result to the cost of NSI reported in Japanese studies (Table 16).

**Table 16:** List of Japanese studies in cost of needlestick injury.

Author	Year	Population	Cost of NSI	Source	Injured	PEP	Follow ups
Tanaka et al [76]	1996	HBV	¥116,270 (US$1,068.86)	Not included	Tested	HBIG (2,000IU)	Not reported
Urano et al [77]	1997	Not reported (no infection?)	¥97,307 (US$804.25)	Not included	Tested	Not provided (HBsAb+)	x5
Yukawa [51]	2005	Not reported (no infection?)	¥49,410 (US$448.29)	Not included	Tested*	Not included	x4
Matsui et al [78]	2007	Not reported (no infection?)	¥93,842 (US$796.93)	Not reported	Not reported	Not included	Yes (no detailed information)
Nishiuchi [79]	2013	No infection, HBV, HCV, HIV (male/female)	¥23,700 (US$242.84)(no infection), ¥119,310 (US$1,222.49) (HBV), ¥37,300 (US$382.19) (HCV), ¥189,570 (US$1,942.40) or ¥190,620 (US$1,953.15) (HIV male/female)	Tested	Tested	[HBV) HBIG (2,000IU) and x3 vaccine [HIV] PEP	x1 (no infection) or x3 (infection)

NSI: needlestick injury, HBIG: hepatitis B immune globulin; PEP: post exposure prophylaxis

*not explicitly stated–based on our best guess

US$ in 2018 value

Differences in outcomes between our study and the previous studies could be explained by different populations and levels of resource use, e.g. Tanaka [76] took into account HBV contaminated NSI only, whilst Urano [77] applied five follow-ups for all cases, rather than biannual fee regulation changes. Nishiuchi [79] used the same number of follow ups as in our study. Cost per NSI was reported as ¥23,700 (US$242.84 in 2013 value) vs. ¥35,510 (US$321.58 in 2018 value) from a payer perspective for infection free NSI, ¥119,310 (US$1,222.49 in 2013 value) vs. 99,936 (US$905.03 in 2018 value) for HBV contaminated case, ¥37,300 (US$382.19 in 2013 value) vs. ¥56,250 (509.41 in 2018 value) for HCV contaminated case, and ¥189,570/¥190,620 (US$1,942.40/1,953.15 in 2013 value) (male/female) vs. ¥254,262 (US$2,302.62 in 2018 value) for HIV contaminated NSI, respectively. Although details of the cost breakdown are not provided in Nishiuchi’s report, the overall trend is very similar to our outcomes except for HBV related injury.

### Sensitivity analysis

Our sensitivity analysis shows the magnitude of impact of key parameter uncertainties on outcomes. We firstly looked at number of NSIs. Although EPINet is well designed and widely used in Japanese medical society, use of this system is not mandatory at National level. Therefore, the EPINet report is not fully representative of Japanese NSIs. Reporting rate is another challenge in estimating number of NSIs, with significant variation between hospitals. We applied discrete parameter values extracted from Japanese multi-centre studies in the sensitivity analysis, ranging from 4.0 to 6.2 NSIs/100beds and 12% to 34.7% for reporting rate, but generalizability of those estimates was not well discussed. Number of NSI/100beds could also vary depending on number of and type of needles used. Suzuki [80] reported a reduction in NSIs by using safety devices. Arise et al [11] reported 110 hospitals (69.2%) and 99 hospitals (62.3%) have already introduced safety equipped butterfly needles and indwelling needles respectively, from a survey of 159 hospitals in 2010, although the proportion of those safety devices in actual use were not reported. We assumed multi-centre studies such as Kimura [30] include use of safety devices at time of survey. Kidouchi [29] clearly mentioned that their setting had started to use safety needles before or during the study period. We assume a small, but unquantifiable, percentage of safety needles are included in our study.

Turning to the labour compensation scheme, we found that the scheme is not well designed to cover the cost of post NSI management, although there are some recommendations for infection contaminated NSI, i.e. HIV post exposure prophylaxis and HCV treatment. Coverage of initial laboratory testing is not clearly stated in the scheme, and therefore practice is not standardized e.g. some hospitals only recommend laboratory testing at time of injury when there is no record of blood test results in a certain period. Some hospitals pay the initial laboratory test fee while others require the injured HCW to pay out-of-pocket. As a result, only 62.3% of 159 hospitals in 2010 applied to the labour compensation scheme for reported NSI cases [11]; Kimura [30] also reported only 21% applied to the scheme. We therefore recommend that a comprehensive and easy to follow compensation scheme as part of a guideline of post NSI management would provide a significant and valuable first step toward providing a safe working environment for HCWs in the healthcare setting.

### Study limitations

Our study has several limitations. First of all, we hypothesized 100% reporting rate and 100% adherence in treatment and follow ups. Although we found some estimates for reporting rates, no study reported the outcome of unreported cases. In addition, some HCWs may visit a clinic during non-work hours whilst others may only notice acquired infectious disease later in life. We were not able to account for those uncertain events and therefore decided to assume all cases are reported at time of injury, and that prophylaxis and treatment against infectious diseases are applied in 100% of cases by following the latest recommendation. Furthermore, although two single institutional studies reported drop-out rates between 18% and 36% [46] [58], they were not generalizable. Therefore, as it is not possible to estimate the number of drop-outs that are followed up, adherence rates were assumed to be 100%.

Secondly, despite the emotional distress associated with NSI being one of the biggest concerns from healthcare professionals’ perspective, the totality of its burden, including legal compensation and absenteeism, or unemployment were not included in our study, as emotional distress associated with NSI is not covered by Japan’s labour compensation scheme and we could not differentiate counselling visits due to NSI from visits unrelated to NSI in the national report of medical resource use.

Thirdly, instead of applying different level of resource utilization dependent on HBV status, e.g. presentation of HBs and HBe antigen and antibody, since the rate of HBV transmission rate is different, we have decided for simplicity to apply an overall figure for HBV, as the cost of HBV related NSIs accounts for less than 1% of total cost of NSIs. Taking the more complex approach would have only marginally increased the base case estimate accuracy. For the same reason, we limited the time horizon of our study to one year, by excluding burden of long-term treatment in HBV, HCV and HIV. Other infectious diseases, such as typhoid and HTLV-1 were also not considered. Although some studies included post exposure antibiotics treatment and increased number of follow ups, no guideline recommends use of such additional resources.

Finally, although NSI is also widely reported outside the hospital [81], our study did not include NSIs that happened outside of the hospital setting, such as out-patient clinics or in the community, due to the lack of national level reporting regarding the size and burden of NSI. With increasing numbers of home medical treatment, e.g. insulin therapy, home dialysis and prophylactic injections for haemophilia, NSIs in the community are becoming more widely reported. Tokyo metropolitan government reported 6 NSIs in 2009, 8 in 2010 and 2 in 2011 [82], whilst Tokyo Building Maintenance Association also reported NSI as a potential hazard in their work environment in community [83]. Unfortunately, there is no National level report regarding the size and burden of NSIs in community. We believe despite challenges in detecting NSI in the community, that NSI is becoming a potentially huge hazard in our community.

Despite these limitations, our study is the first study in Japan to estimate the total cost burden of in-hospital NSIs. Our study is updated with new treatment regimen for HCV (2018) and labour compensation scheme for post-exposure prophylaxis for HIV (2010) and HBV (2004). We hope that this study will guide decision makers and encourage further action to set up a safe work environment for HCWs.

## Conclusion

Total cost burden of in-hospital NSIs was estimated as ¥33.4 billion (US$302 million) and cost per NSI was estimated as ¥63,711 (US$576.97), which are comparable with previous studies. The outcome is largely dependent on the estimated number of NSIs. Number of follow ups especially for infection-free cases and prevalence of blood borne disease, especially HCV, are also key drivers in influencing outcomes. One of the biggest challenges in our study was the institutional level variation of post NSI management.

Our study suggests that reducing number of NSIs, e.g. by using safety devices, will reduce the total cost significantly. Development of a clear, comprehensive and easy to follow labour compensation scheme specific to NSI is recommended in order to encourage reporting and increase adherence in post NSI management. Further analysis is suggested by including cost of emotional distress, and NSIs in community with increased application of home medical treatments in Japan.

## Supporting information

S1 Appendix Search strategy. (PDF)

S2 Appendix Inclusion-Exclusion criteria. (PDF)

S3 Appendix Cost of medical resource use. (PDF)

S4 Appendix PRISMA checklist. (DOCX)
