# Supplementary material for: Estimating the national cost burden of in-hospital needlestick injuries among healthcare workers in Japan
Source: PLoS One. 2019 Nov 7;14(11):e0224142. doi: 10.1371/journal.pone.0224142 (PMC6837393; doi:10.1371/journal.pone.0224142)
Supplement: S1 Appendix — (PDF) [file pone.0224142.s001.pdf]

S1 Appendix: Search strategy

Database search
PubMed
Key Words: (((needlestick*[Title] OR needle-stick*[Title] OR sharp*[Title] OR 'body fluid exposure'[Title] OR 'blood exposure'[Title])) AND (cost*[Title/Abstract] OR burden*[Title/Abstract] OR 'economic evaluation'[Title/Abstract] OR QALY*[Title/Abstract] OR Societal[Title/Abstract] OR Utilit*[Title/Abstract] OR 'resource use'[Title/Abstract]))
Country: no limitation
Language: English and Japanese
Year of Publication: no limitation
Search Date: 21st October, 2018
Ichushi
Key Words: 針刺し[TI]
Country: Japanese study
Language: English and Japanese
Year of Publication: no limitation
Search Date: 21st October, 2018

Grey search
1. Journal of Exposure; the International Healthcare Worker Safety Center
2. The Journal of Japan Society for Health Care Management (2001~2002); International Association of Risk Management in Medicine
3. The database of funded studies; the Ministry of Health, Labour and Welfare

*following journals/academic societies are confirmed to be included in either PubMed or Ichushi

- Value in Health; The International Society of Pharmaceutical and Outcomes Research
- Value in Health regional issues; The International Society of Pharmaceutical and Outcomes Research
- International Journal of Technology Assessment in Health Care; Health Technology Assessment International
- Japanese Society for Infection Prevention and Control
- The Japanese Association for Infectious Diseases
- The Japanese Journal of Quality and Safety in Healthcare; Japanese Society for Quality and Safety in Healthcare
- Japan Society of Clinical Safety
- Healthcare and Safety; Japan Society of Clinical Safety
- The Journal of Japan Society for Health Care Management (2003~); International Association of Risk Management in Medicine
- The Journal of Japan Society for Health Care Management; International Association of Risk Management in Medicine
- Japanese Nursing Association (series of publications)
- Journal of Japan Academy of Nursing Science; Japan Academy of Nursing Science
- Proceedings of the Academic Conference, Japan Academy of Nursing Science; Japan Academy of Nursing Science
- Journal of Japan Operative Nursing Academy; Japan Operative Nursing Academy
- Japanese Society of Medical Instrumentation
- Japanese Journal of Infectious Diseases
- Infection Control;

Snowball search
By visiting all relative studies identified in the articles went through full-text review
