# Supplementary material for: Estimating the national cost burden of in-hospital needlestick injuries among healthcare workers in Japan
Source: PLoS One. 2019 Nov 7;14(11):e0224142. doi: 10.1371/journal.pone.0224142 (PMC6837393; doi:10.1371/journal.pone.0224142)
Supplement: S2 Appendix — (PDF) [file pone.0224142.s002.pdf]

S2 Appendix: Inclusion-Exclusion criteria

	Criteria	Model	Parameter
a	Population	NA	Limited population, such as dental clinic, operation room, dialysis clinic/room, student, infection related NSI, insulin NSI
b	Topic	other than needlestick injury, sharp injury and body fluid exposure	other than needlestick injury, sharp injury and body fluid exposure
c	Country	NA	Other than Japan
d	Method	case study, discussion, editorial	case study, discussion, editorial
e	Outcome	no cost	No report in #NSI, %NSI, % reporting, Infection status, and resource utilization
l	language	Other than English and Japanese	Other than English and Japanese
