# Supplementary material for: Estimating the national cost burden of in-hospital needlestick injuries among healthcare workers in Japan
Source: PLoS One. 2019 Nov 7;14(11):e0224142. doi: 10.1371/journal.pone.0224142 (PMC6837393; doi:10.1371/journal.pone.0224142)
Supplement: S3 Appendix — (PDF) [file pone.0224142.s003.pdf]

S3 Appendix: Cost of medical resource use incurred during follow-up and treatment

					at injury	all	HBV	HCV	HIV	HCV treatment
	Number of follow ups					1	3	3	3	1
**Category**	**Name of fee**	**2018 point**	**NHI price***	**labour compensation price***						
**Consultation fee**	First consultation fee	282	¥2,820	¥3,760	1					
	Consultation fee	72	¥720	¥1,390		1	3	3	3	1
	Out-patient fee	52	¥520	¥624						1
	Education fee (labour compensation only)	NA	¥0	¥920		1	3	3	3	
	Documentation fee (labour compensation)	NA	¥0	¥2,000	1					
	Emergency consultation fee (labour compensation)	NA	¥0	¥1,250						
	Brushing	91	¥910	¥1,092						
**Test**	Collecting blood	16	¥160	¥192	1	1	3	3	3	1
	Emergency test	10	¥100	¥120	1					
	Bio chemical test	112	¥1,120	¥1,344	1	1	3	3	3	1
	Reading the result of bio chemical test	144	¥1,440	¥1,728	1	1	3	3	3	1
	Hepatitis related test	290	¥2,900	¥3,480	1	1	3	3	3	1
	HIV test	118	¥1,180	¥1,416	1	1	3	3	3	
	HTLV-1 test	86	¥860	¥1,032	1	1	3	3	3	
	Syphilis test	15	¥150	¥180	1	1	3	3	3	

	Reading the result of immune test	144	¥1,440	¥1,728	1	1	3	3	3	1
	Test sample management	40	¥400	¥480	1	1	3	3	3	1
**Prophy**	HB vaccine	2,382	¥2,382	¥2,382			3			
	intramuscular injection	20	¥200	¥240			3			
	HBIG	35,322	¥35,322	¥35,322			1			
	NS 100mL	128	¥128	¥128			1			
	intravenous injection	49	¥490	¥588			1			
	HCG test	55	¥550	¥660					1	
	Reading the result of bio chemical test	144	¥1,440	¥1,728					1	
	Truvada®	3,863.6	¥108,181	¥108,181					1	
	Isentress®	1,553.6	¥87,002	¥87,002					1	
		42	¥420	¥1,008					2	
**Treatment**	HCV treatment		¥3,971,527	¥3,986,256						1
TOTAL (2018 price)					¥12,570	¥10,370	¥74,796	¥31,110	¥229,122	
TOTAL (labour compensation)					¥17,460	¥13,890	¥85,574	¥41,670	¥241,256	
test and visit for HCV treatment (2018 price)										¥8,700
test and visit for HCV treatment (labour compensation)										¥10,966

*1 reimbursement point = ¥10 for payers perspective (under national health insurance scheme) or ¥12 for societal perspective (under labour compensation scheme)
